# Mr. Roberton's Plan of Medical Police

**Published:** 1808-03

**Authors:** John Roberton


### *To the Editors of the Medical and Physical Journal.*

Gentlemen,

THE following letter was printed, for the purpose of obtaining the Reports to which it alludes; but as the Post Office regulations of this country, unfortunately, do not permit these reports to be franked, and as the receipt of numerous Communications would be enormously expensive to me, I take the opportunity of communicating it, through the means of your Journal, to the numerous medical practitioners who are its readers, and shall think myself myself very highly obliged to all those Gentlemen who favour me with such Reports, (free of postage) addressed to me, No. 4, St. James's Street, Edinburgh. I shall mysely give a detailed Report of the permanent sources of the diseases of Edinburgh, and shall be happy publicly to acknowledge every communication.

This proposition was intended to be the commencement of a series of similar ones; and I am happy to see that the College of Surgeons, of this place, have adopted the principle.—From them we have a right to expect much.

I am, &c.

JOHN ROBERTON.

---

" SIR,

" THE importance of the subject on which I am about to address you, has induced me to forward a copy of this letter to persons of the greatest respectability throughout the country, and, among others, I have thought it my duty to transmit one to you.

" Ever since the publication of my Treatise on the use of Cantharides in Gleet, Leucorrhœa, and Obstinate Sores, I have employed myself in collecting materials for a work, which, if my success answers my expectations, I shall lay before the public, under the title of " A PLAN OF MEDICAL POLICE."

" I am well aware, that the general arrangement of such a plan as it may be necessary to follow upon this interesting subject, is replete with difficulty; and that the particular one which I have here indicated, may, in many points, be susceptible of improvement; but, as it will ultimately be productive of numerous benefits, as far as health is concerned, it must become a subject of great national importance. If, indeed, even the life of a single individual is preserved by my exertions, I shall be contented, in the expectation that others, improving the principle, may at length bring my plan to a state of greater perfection.

" Many of my opinions on this subject must long have been familiar to every one; as several of those causes which first induced me to think seriously about it, have been acknowledged sources of disease, from the earliest periods of history; but, so far as I am acquainted with the writings of former times, no author has been fortunate enough, either from incorrect statements of facts, or from deficient or erroneous modes of reasoning, to induce the

Legislative

Legislative Body of this country to adopt a general systematic plan for their prevention.

" The principal general sources of disease in this, and perhaps in every other country, I believe, with very few exceptions, to exist in external, and, for the most part, removable causes; but, from our familiarity with numberless circumstances which are unquestionably injurious to our comforts, and even destructive to our constitutions, we, in the common bustle of life, insensibly so overlook them, as scarcely ever to regard them in a just point of view. Many are willing to allow, that these sources are injurious to their comforts, but few believe them capable of ruining their constitutions.

" The fenny counties of England, and other swampy grounds, most of which, though drainable, are suffered to exist, form well known sources of diseases; and that uncleanness and filth which, in every part of our island, obtrudes itself upon our notice, contributes no small share to the propagation of them.

" The local situation and the internal structure of dwelling-houses, also constitute another great source of them.

" In earlier times, both these causes must have greatly contributed to their propagation and frequency; and although history informs us of uncommon feats of strength performed by individuals in former times, yet from this we can draw no conclusion respecting the health of ancient nations in general. For I believe that, in every part of the world, the greatest proportion of its inhabitants have passed their lives in obscurity, and although employed in the most useful pursuits, their names and their diseases are equally unknown to us; they are only rare occurrences, magnified by tradition, that have been transmitted to us.

" *But whatever diseases are found to arise from these, or indeed from any general cause—any derangement of natural objects, I am convinced they may, even in the present imperfect state of Physics, though with difficulty, be wholly, or for the most part removed.*

" If the poor alone were sufferers from the operation of such causes, the wonder would not be so great that nothing has been done for their prevention; but the rich are often equal sufferers. I do not mean to say that they are so in every respect. They are not like those cold and hungry wretches, who in general experience a paroxysm of joy only when in a state of intoxication, and who are obliged to hovel together in obscure and cheerless garrets, hardly hardly protected even from the severity of the winter's storm, or are compelled to groan out their existence in loathsome cellars, and to breathe an atmosphere impregnated with every quality that is destructive of human life; where, in general, moral principles are as much corrupted, as physical powers are destroyed; where the young of our own species are at once tainted with disease, and trained from infancy in the exercise of every thing that is vicious, who, if they had been placed in more favourable physical circumstances, and exposed to more favourable moral opportunities, might possibly have done honour to humanity.

" Thousands of parents, in the situations here alluded to, whose principles, both moral and religious, have (if I may use the term) been starved out of them, distort the limbs, or destroy the eyes of their infants, that they may thus be exposed in the publie streets, and rendered objects for exciting the pity, and means of extorting the money of passengers.

" While the community, in general, can only survey the black outline of human distress, without even suffering themselves to attach it that pity which is really its due, Medical Practitioners, particularly in large and populous cities, are daily, while prosecuting the duties of their profession, obliged to witness indescribable scenes of misery and wretchedness.

" That the removal of many *such nuisances* as I have mentioned above—*the causes of all these calamities*, should never have been thought of, particularly in Great Britain, where scientific research constitutes so prominent a feature of the natural character, has to me often been a matter of surprise and wonder. The adoption of such a plan would not only contribute to the happiness of individuals, but to the increase of the public revenue; as the healthy state of a country is one of the greatest—is indeed the first cause of its prosperity and affluence.

" The wealth of all nations arises from their quantity of productive labour; and therefore whatever proportion of unproductive individuals exist in any country, not only is a similar proportion of wealth lost to it, but an additional proportion of its wealth is destroyed. Mr. Malthus has proved, that an increase even of active population would, at the present moment, be prejudicial to Great Britain. How infinitely more so must an increase of inactive population be, not only as affording no aid to the revenue from the taxation of labour or of income, and as being burdensome some to their respective parishes, but as more completely exceeding the means of subsistence!! A more detailed view of this proposition will appear in the London Medical and Physical Journal.

"If therefore, SIR, you will, as soon as may be convenient for you, send me A STATEMENT OF THE PREVAILING DISEASES OF YOUR PART OF THE COUNTRY (alluding chiefly to such as may arise from any general cause), AND OF WHAT GENERAL CAUSES THEY SEEM TO EXIST; such as fens, marshes, ill-constructed houses, &c.; or if you know any person, medical or otherwise, who may be able to contribute to this undertaking, you will, I am convinced, do your country an infinite service, and confer an essential favour on,

"Sir,

"Your most obedient humble Servant,

"JOHN ROBERTON."

*Edinburgh, Nov. 6, 1807.*

---

### *To the Editors of the Medical and Physical Journal.*

GENTLEMEN,

